# Supplementary material for: Cell-vision fusion: A Swin transformer-based approach for predicting kinase inhibitor mechanism of action from Cell Painting data
Source: iScience. 2024 Jul 15;27(8):110511. doi: 10.1016/j.isci.2024.110511 (PMC11340608; doi:10.1016/j.isci.2024.110511)
Supplement: Document S1. Figures S1–S11 and Tables S1‒S3 [file mmc1.pdf]

iScience, Volume *27*

Supplemental information

Cell-vision fusion: A Swin transformer-based approach for predicting kinase inhibitor mechanism of action from Cell Painting data

William Dee, Ines Sequeira, Anna Lobley, and Gregory Slabaugh

SUPPLEMENTARY INFORMATION

SUPPLEMENTAL ITEM TITLES

S1. Shapley importance values per class
S2. Modality ablation study
S3. U2OS-specific kinase inhibitor class selection
S4. Image file formats
S5. Quality control
S6. Mechanism of action - Class distribution
S7. Plate-wise, channel-wise standardization overview
S8. Fusion performance compared to compound clinical phase
S9. Predicting microscope and data source
S10. Study workflow

S1. Shapley importance values per class

Displayed in Figure S1 are the top ten features for predicting each mechanism of action (MOA) when using an XGBoost model, ranked according to Shapley importance value.

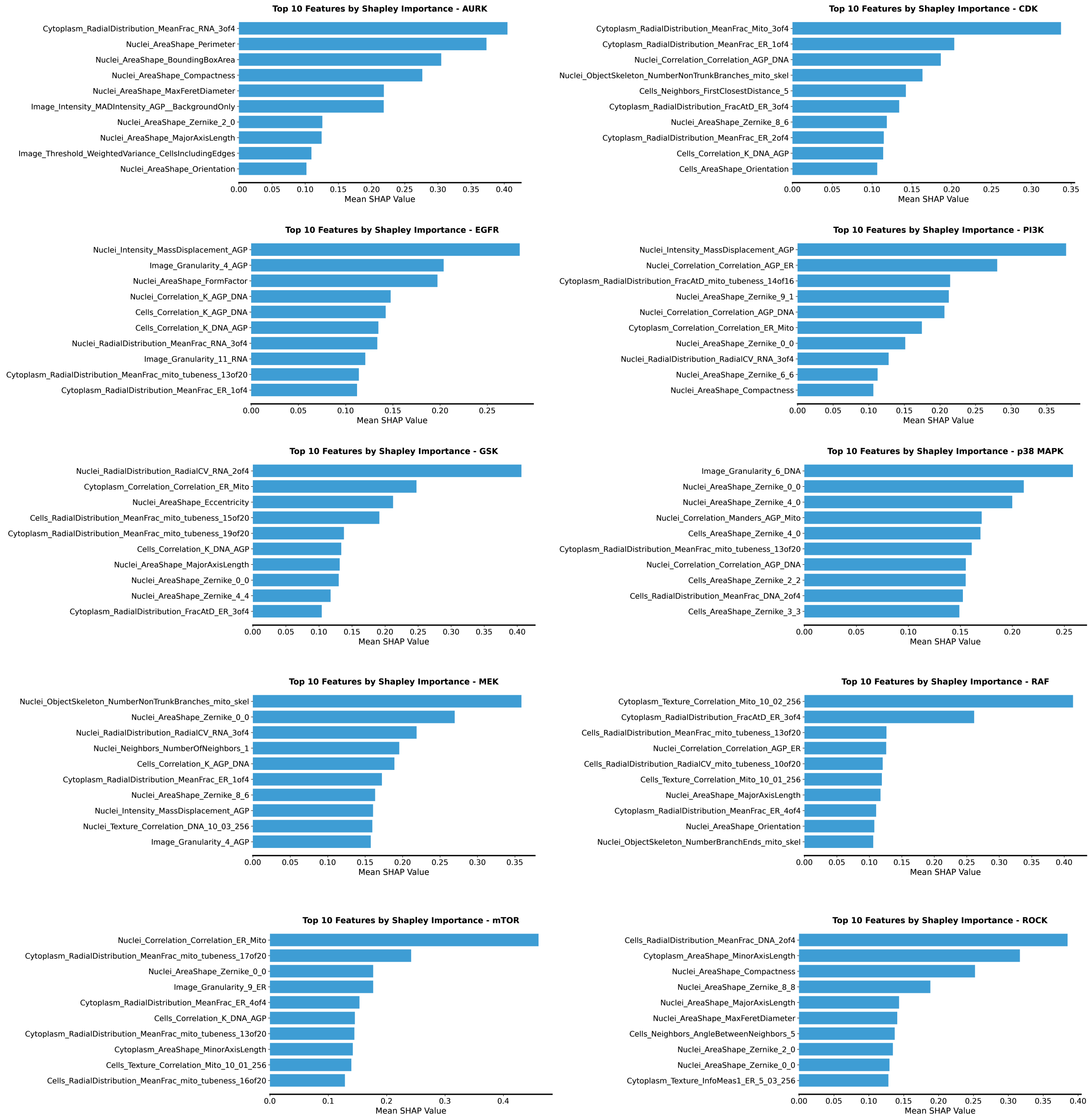

Supplemental Information, Figure S1. Top ten Shapley-ranked features for each class, related to Figure 3.
Shapley importance values for the top ten ranked features for predicting each MOA class in the kinase inhibitor dataset.

S2. Modality ablation study

Table S1 contains ablation study results whereby models were evaluated using combinations of two of the data modalities present – from images, image-based CellProfiler data and compound structures. The results show that the proposed combination of all three modalities (i.e. Cell-Vision Fusion) performs better than the two-modality approaches in accuracy, F1 score and precision. Excluding the compound structure data improved the recall, AUPR and AUC, however there is clearly a trade-off between classification performance and ranking ability (displayed by the lower accuracy, F1 score and precision for this combination).

Compound structure is the least diverse set of data, as it presented at a compound level (i.e. each compound has a particular compound structure), whereas the image-based profile data is added at the well level and the images are at a field level (i.e. sub-well). This lack of diversity in the compound data likely lead to this reduction in ranking ability of the model combinations which included it. Additionally, when compound structure is used alongside only the image data, the combined model suffers from overfitting and the model results are substantially worse. In a larger, more diverse, dataset we would expect the inclusion of compound structure data to be more beneficial, as has been shown in prior research[(1)].

	Five-fold Cross-validated Results (%)					
**Approach**	Accuracy	F1 Score (Macro)	Precision	Recall	AUPR (Macro)	AUC (Macro)
**Cell-Vision Fusion**	**69.79**	**70.56**	**73.40**	69.87	74.12	90.73
**IBP + Compound Structure**	68.75	68.32	70.58	68.54	73.96	91.02
**Images + IBP**	65.00	63.00	65.67	**70.00**	**82.33**	**93.05**
**Images + Compound Structure**	46.88	45.43	52.11	45.67	53.44	82.01

Table S1. Ablation study performed using the different modalities which contributed to the fusion model, related to Table 1.
Cell-Vision Fusion, the fusion method created by this paper, uses all three data modalities - being images, image-based CellProfiler profiles and compound structures converted into Morgan Fingerprints. We have tested above how the model evaluation metrics are affected if only combinations of two modalities are used.

S3. U2OS-specific Kinase inhibitor class selection

DepMap gene TPM values

Gene expression TPM values for the U2OS cell line were downloaded from the DepMap Public 23Q2 gene TPM values using the DepMap U2OS ID "ACH-000364". The broad kinase inhibitor classes found in the JUMP cpg0016 data were then associated with the specific underlying genes found in the DepMap data (Figure S2A) before the expression values were summed and sorted at a kinase inhibitor class level (Figure S2B). This provided an idea of the likely expression levels of the protein kinases found in U2OS cells which would then impact how significant an impact inhibiting them would have on the phenotypic response of the cell to each inhibitor.

Kinase inhibitor U2OS literature

Kinase inhibitor classes matched to cpg0016 data: Phosphoinositide 3-kinase (PI3K), Epidermal growth factor receptor (EGFR), p38 mitogen-activated protein kinase (p38 MAPK), Janus kinase (JAK), Rapidly Accelerated Fibrosarcoma (RAF), Aurora kinase (AURK), Anaplastic Lymphoma Kinase (ALK), Sarcoma kinase (SRC), Rho-associated kinases (ROCK), Mitogen-activated protein kinase kinase (MEK), Glycogen synthase kinase (GSK), Cyclin-dependent kinase (CDK), Vascular endothelial growth factor receptor (VEGFR), Breakpoint cluster region-Abelson kinase fusion protein (Bcr-Abl), Platelet-derived growth factor receptor (PDGFR), Fibroblast growth factor receptor (FGFR), Bruton's tyrosine kinase (BTK), Protein kinase B (AKT) and Mammalian target of rapamycin (mTOR).

From these classes, we considered which ones were appropriate for prediction, given the cpg0016 experiments had been conducted only using the U2OS cell line. Due to the cell type, there were multiple classes which will likely not produce a distinguishable phenotypic response that a model would be able to interpret. The brief rationale for each class is summarized below:

- **Vascular endothelial growth factor receptor (VEGFR):** VEGF action is primarily targeted to endothelial, rather than

epithelial cells[2]. As such, we would not expect to see a significant phenotypic response from this class of inhibitors when administered in U2OS cells which have an epithelial morphology.

- **The Bcr-Abl fusion protein and Bruton's tyrosine kinase (BTK):** Both are primarily activated/expressed in hematopoietic cells[3,4].

- **Platelet-derived growth factor receptor (PDGFR) and Fibroblast growth factor receptor (FGFR) Inhibitors:** PDGFR and FGFR are primarily expressed in mesenchymal cells, including fibroblasts, smooth muscle cells, and pericytes, but not in epithelial cells[5].

- **Janus Kinase (JAK):** The JAK-STAT pathway is triggered by the binding of cytokines to their respective receptors. Unlike immune cells, epithelial cells typically do not express high levels of cytokine receptors. Furthermore, JAK inhibitors, especially earlier generations, are known to exhibit low selectivity, leading to wide-ranging off-target effects[6]. Due to this, including the JAK class may introduce a lot of noise into the dataset, making it more difficult to classify other, more selective, classes. Despite this, Janus kinases play a central role in inflammation and therefore their impact can be significant across different cell types.

Whilst PI3K and EGFR demonstrated relatively low gene expression values (Figure S2B), the literature review highlighted the importance of the pathways they are directly involved in, as well as the fact that the upstream nature of these two classes of inhibitors should mean that they demonstrate phenotypic responses which will be appropriate to model.

A

```
ki_tgt_dict = {
    'PI3K': 'PIK3CA|PIK3CB|PIK3CD|PIK3CG',
    'EGFR': 'EGFR',
    'p38 MAPK': 'MAPK11|MAPK12|MAPK13|MAPK14',
    'JAK': 'JAK1|JAK2|JAK3|TYK2',
    'RAF': '^ARAF|BRAF|^RAF1',
    'AURK': 'AURKA |AURKB|AURKC',
    'ALK': 'ALK ',
    'SRC': '^SRC |LYN |FYN|YES1|FGR|BLK|HCK|LCK',
    'ROCK': 'ROCK1|ROCK2',
    'MEK': 'MAP2K1|MAP2K2',
    'GSK': 'GSK3A|GSK3B',
    'CDK': 'CDK1 |CDK2 |CDK4 |CDK5 |CDK6 |CDK7 |CDK9 ',
    'VEGFR': 'FLT1|^KDR|FLT4',
    'BCR-ABL': '^ABL1 |^ABL2 |BCR',
    'PDGFR': 'PDGFRA|PDGFRB',
    'FGFR': 'FGFR1 |FGFR2|FGFR3|FGFR4',
    'BTK': '^BTK',    # '^' added to exclude IBTK
    'AKT': 'AKT1 |AKT2|AKT3',
    'mTOR': '^MTOR '
    }
```

B

k_inhib	exp_val
MEK	6.034046
CDK	6.030705
GSK	5.909385
AKT	5.856681
AURK	5.759314
mTOR	5.100557
RAF	5.077632
ROCK	4.744178
FGFR	4.057675
p38 MAPK	3.689417
BCR-ABL	3.515022
JAK	3.309181
PDGFR	3.305077
SRC	2.444012
EGFR	2.163499
PI3K	2.159123
VEGFR	1.168495
ALK	0.201634
BTK	0.124328

Supplemental Information, Figure S2. Mapping of kinase inhibitor classes to DepMap data, related to Figure 1.
A) Shows a dictionary used to map kinase classes identified in the cpg0016 dataset to the genes within the DepMap expression TPM value data. b) displays the value responses for each gene amalgamated at a kinase inhibitor class level, implying that classes that inhibit proteins associated with highly expressed genes (i.e. MEK, CDK inhibitors) will likely show a more significant phenotypic response in U2OS cells compared with those classes where associated gene expression is low (i.e. ALK, BTK).

S4. Image file formats

Table S2 contains a summary of the image file formats for each contributing source to the cpg0016 dataset. The information was extracted from image files downloaded from the cpg0016 AWS s3 bucket.

Technical Specifications

Measure	Source 1, 3, 4, 9	Source 2, 5	Source 6	Source 7	Source 8	Source 10	Source 11
**Resolution**	1,080 x 1,080	996 x 996	970 x 970	1,280 x 1,080	1,024 x 1,024	1,000 x 1,000	1,080 x 1,080
**Bit Depth**				16			
**Compression**	LZW			Uncompressed			LZW
**Microscope**	Opera Phenix	CV8000	CV8000	CV7000	ImageXpress	CV8000	Operetta

Table S2. Technical specifications of images produced by the different cpg0016 data sources, related to STAR Methods - Data acquisition.
Includes image resolution, bit depth, and compression as well as the different microscope names.

S5. Quality control

Columns from the CellProfiler IBP feature sets were extracted and associated to three different quality control measures - blur, saturation and focus. In total, 25 blur features, 10 saturation features and 25 focus features were identified. These features were min-maxed normalized and plotted to show their distribution throughout our kinase inhibitor dataset. Figure S3 shows the distribution of the blur scores, coloured according to the source where the datapoint was produced.

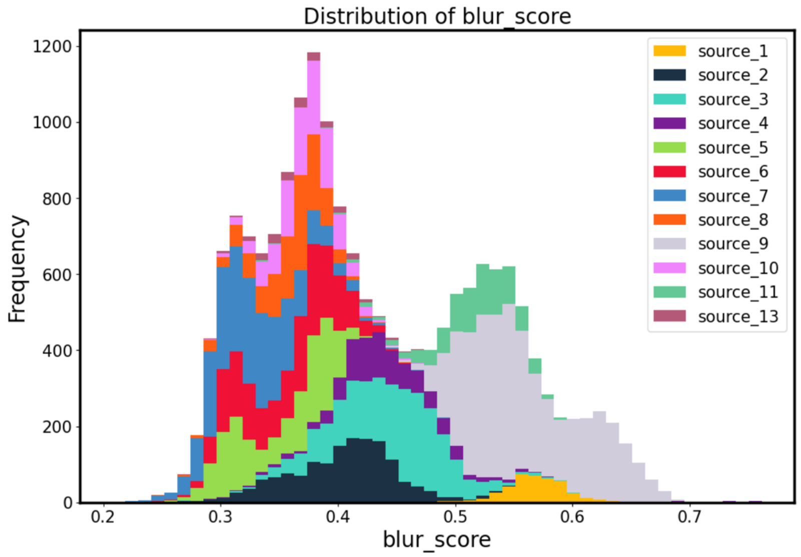

Supplemental Information, Figure S3. Distribution plot showing the levels of blur present in the dataset images, related to STAR Methods - Quality control.
A stacked histogram of the composite, normalized blur scores for each datapoint within the kinase inhibitor dataset, coloured according to which source produced the data. Blur scores were calculated as a mean score from all CellProfiler features associated with the image quality control metric blur, being "ImageQuality_Correlation", "ImageQuality_PowerLogLogSlope" obtained from the CellProfiler manual.

The saturation and focus scores had to be transformed to be comparable to the blur scores. This was due to the fact that the saturation scores were highly skewed, whilst the focus scores were calculated such that a lower score meant lower quality (i.e. low focus), compared to blur scores where a low score meant higher quality (i.e. low blur). Therefore, the saturation scores were transformed into a normal distribution using sklearn's QuantileTransformer module, whilst the focus scores were inverted (Figure S4). On inspection of the images, however, both highly and lowly saturated images were recognised to be poor quality. To combat this, a tanh transform was applied to the saturation scores to ensure all images scoring highly in this metric were reflective of a low quality image (Figure S5).

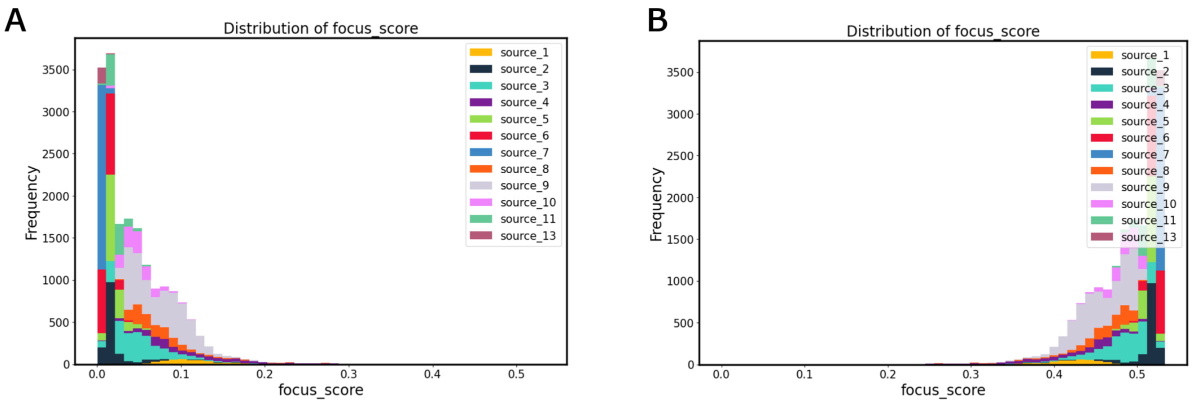

Supplemental Information, Figure S4. Distribution plot showing the levels of focus present in the dataset images, related to STAR Methods - Quality control.

A) shows the composite mean focus scores for each datapoint before the data was inverted. B) shows the data post-inversion, where a high focus score now equates to a low quality (low focus) image. This transform was performed so the same cutoff could be applied to all three metrics to exclude low quality images. The CellProfiler focus measures used were "ImageQuality_Focus", "ImageQuality_LocalFocus".

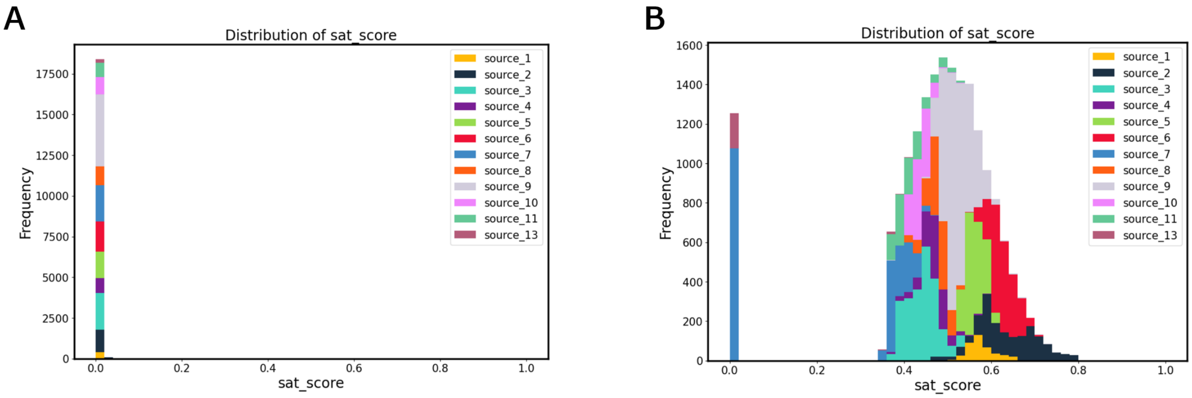

Supplemental Information, Figure S5. Distribution plot showing the levels of image saturation present in the dataset, related to STAR Methods - Quality control.

A) shows the composite mean saturation scores for each datapoint before the data was inverted. B) shows the data after transformation to a normal distribution, followed by a tanh transformation, where a high saturation score now equates to a low quality (lowly or highly saturated) image. This transform was performed so the same cutoff could be applied to all three metrics to exclude low quality images. The CellProfiler saturation measures used were "ImageQuality_PercentMaximal", "ImageQuality_PercentMinimal".

After transforming these measures, they were used to exclude images from selection within our dataset. We applied a 90th percentile cutoff to each metric, ensuring no datapoints, where images which ranked above this threshold for any of the metrics, were included. Figure S6 shows examples of images which were excluded based on this criteria.

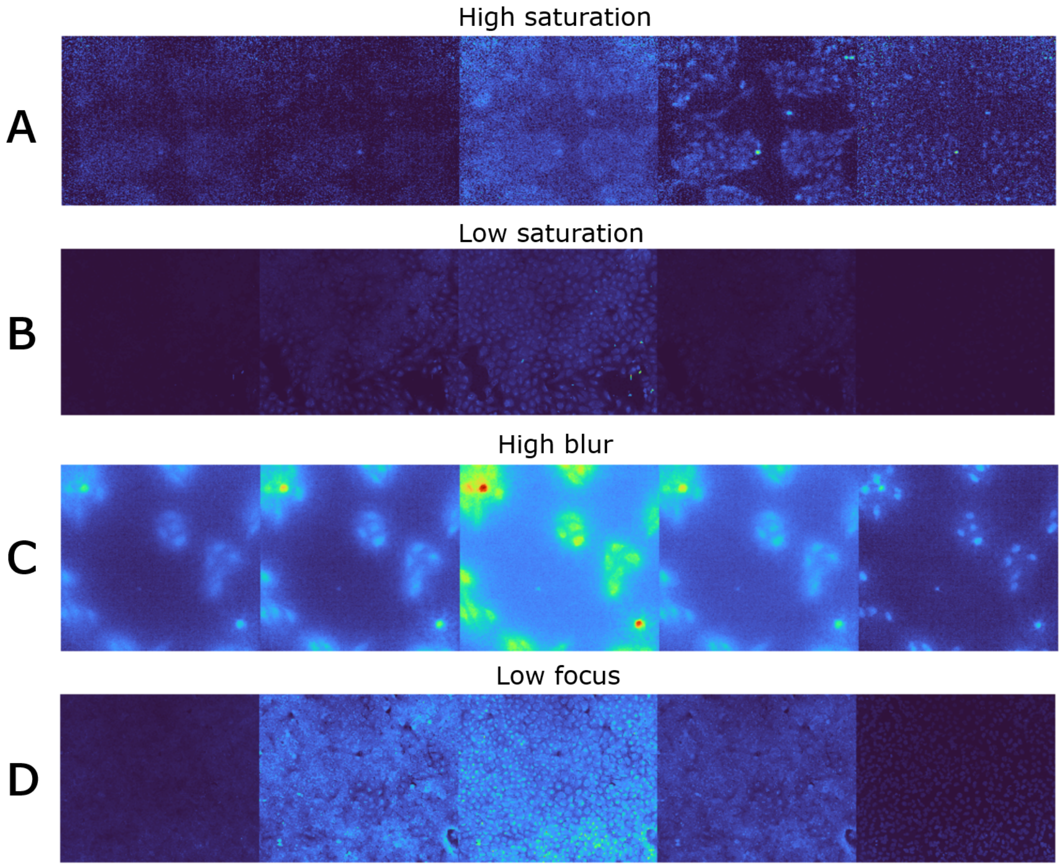

Supplemental Information, Figure S6. Example images excluded post quality control measures, related to STAR Methods - Quality control.
Example five channel Cell Painting images of datapoints which were excluded based on the quality control metrics defined above, showing examples of: A) high saturation B) low saturation C) high blur and D) low focus. Images are plotted using the 'turbo' colourmap to make the components more clearly visible.

S6. Mechanism of Action - Class Distribution

Figure S7 shows the distribution of kinase inhibitor compounds according to both the source where the experiments took place (S7 - A, B) as well as the microscope used to take the images (S7 C,D). These visualizations were used to inspect whether there was any particular clustering of compound mechanism of action according to either of these two major confounding factors. While there is only one sample in the dataset from Source 1 (an Aurora Kinase Inhibitor), and Sources 4 and 5 are over-indexed to the Cyclin-dependent kinase class, overall there is very little clear clustering observed in the below distribution plots which could lead to leakage during model training. Generally class samples are spread across multiple sources and microscopes within the data.

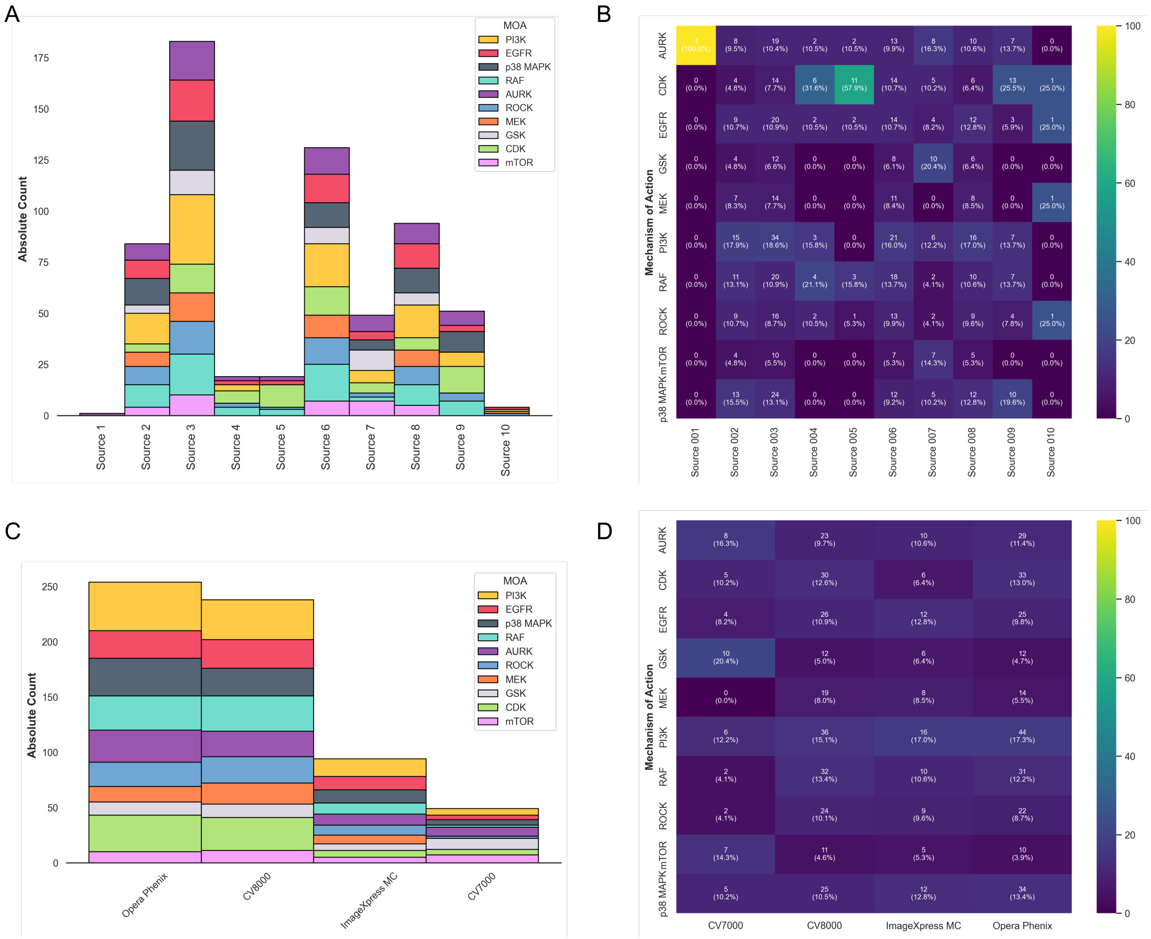

Supplemental Information, Figure S7. Distribution of dataset kinase inhibitor classes across two different contributors to batch effects - originating source and microscope, related to Figures 4 and 5.

A) stacked histogram showing the absolute count of number of class-specific samples found across the different sources in the dataset. B) heat map showing the relative percentage of each class as a proportion of all class samples found in each source (the absolute count is also shown). C) absolute count histogram of class-specific images taken with one of four different microscopes. D) heat map showing the relative percentage of images of each class taken with each microscope.

S7. Plate-wise, channel-wise standardization overview

Figure S8 shows an overview of how PCS is performed on the cpg0016 data to extract the standardization metrics, either median and median absolute deviation or mean and standard deviation, from the positive or negative control samples on each plate.

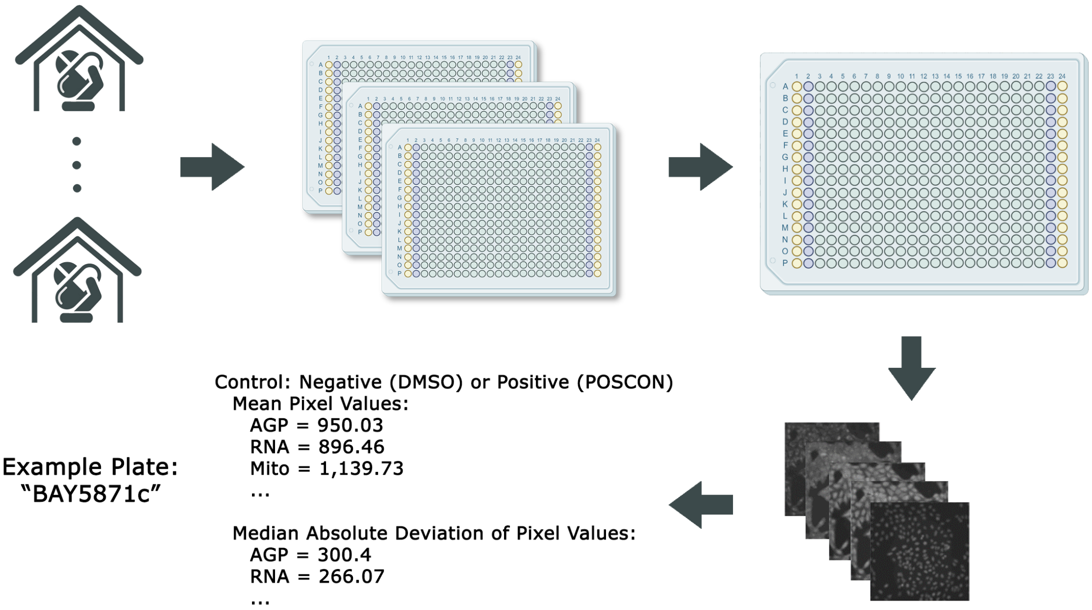

Supplemental Information, Figure S8. Overview of the PCS process, related to STAR Methods - Raw images: Normalization and standardization.
Images of either the positive or negative (DMSO) control samples are downloaded from each experimental plate, and the median, mean, median absolute deviation and standard deviation of pixel values are extracted for each channel. These are then aggregated to a plate-level representation of the control response and then these representations can be used to standardize the data from the experimental wells.

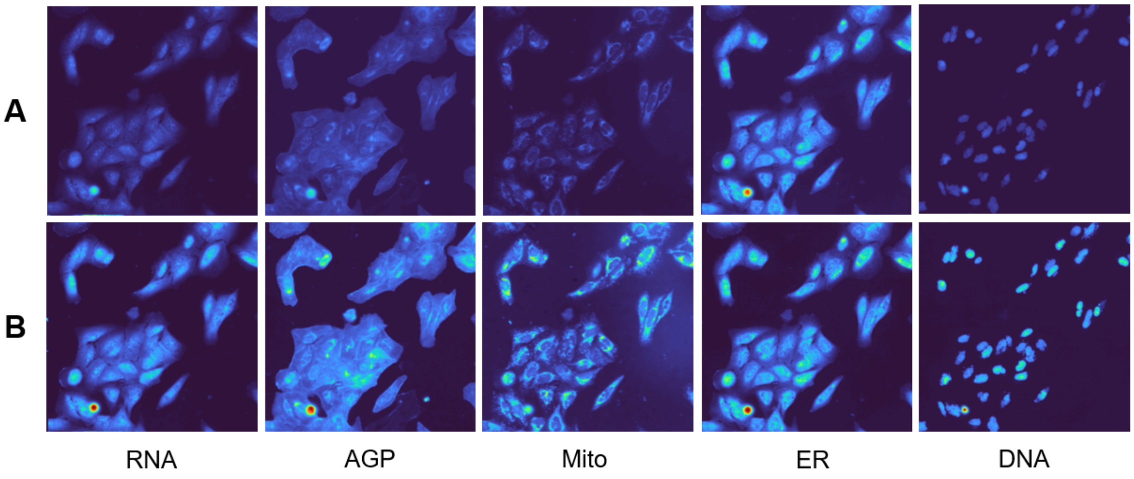

Supplemental Information, Figure S9. Applying PCN Normalization and PCS Standardization to a Cell Painting Image, related to STAR Methods - Raw images: Normalization and standardization.
A comparison between (A) non-normalized images from one field of view taken across the five cell painting channels (RNA, AGP, Mito, ER and DNA) and (B) the same images after undergoing PCS and PCN. Images are displayed using the "turbo" colormap from the Matplotlib package, to make the individual components more clear.

S8. Fusion performance compared to compound clinical phase

The Drug Repurposing Hub and ChEMBL data contain clinical phase information, reflecting how far along in the clinical development pipeline each compound was at the point of inclusion in the dataset. In Figure S10 we compare how this clinical phase impacts model performance, noting that generally as compounds get further along in development they are more accurately identified by the CVF Fusion model. This potentially reflects the fact that drugs which have been launched are more likely to effectively impact their intended target with less off-target effects compared to compounds which are still at the preclinical stage.

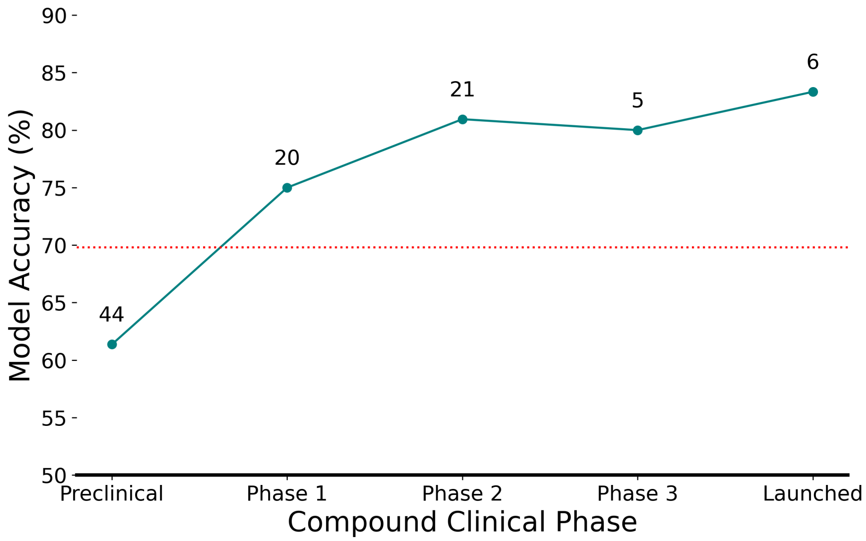

Supplemental Information, Figure S10. CVF model accuracy plotted for compounds grouped according to their clinical phase of development, related to STAR Methods - Analysis and evaluation.

The points are labelled with a number, reflecting the amound of compounds in that phase in the dataset, i.e., there are 44 in preclinical development out of 96 total in our data. The red dotted line denotes the overall CVF performance achieved, showing that for all compounds at Phase 1 or later, the CVF model classifies them with greater than average performance.

S9. Predicting microscope and data source

Table S3 shows results when the SwinV2 model was trained to predict either the source where the data was produced or the microscope used to record the image (as opposed to predict compound mechanism of action). This test was run to observe the impact that the two approaches to normalization and standardization of the image data had on the ability of the model to predict the main confounding factors. The results show that the model is very adept at classifying both microscope and source before any efforts were made to counteract batch effects. Using five-fold cross-validation the model was 99.69% accurate at predicting which microscope was being used and 98.11% accurate at predicting the source. After we applied both plate-wise, channel-wise standardization (PCS) and per-channel normalization (PCN), the model accuracy dropped to 85.98% for microscope and 84.41% for source prediction. This provides evidence that the measures implemented in this paper are, to some extent, reducing the obscuring batch effects present in the data, allowing the model to focus on the biological signal to a greater degree. These results are backed up by the results in the main report.

	Five-fold Cross-validated Results (%)		
**Batch Correction Method**	Prediction	Accuracy	F1 Score (Macro)
**None**	Source	98.11	97.97
**PCN**	Source	92.44	91.63
**PCN + PCS**	Source	**84.41**	**83.70**
**None**	Microscope	99.69	99.69
**PCN**	Microscope	97.80	97.78
**PCN + PCS**	Microscope	**85.98**	**86.04**

Table S3. Image normalization and standardization ablation study, related to STAR Methods - Raw images: Normalization and standardization. Ablation study performed by removing either per-channel normalization (PCN), plate-wise, channel-wise standardization (PCS) or both during model training when the SwinV2 model was used to predict either the data source or microscope used.

S10. Study workflow

Figure S11 includes an overview of the workflow used in this study. Initially the JUMP dataset metadata is combined, before MOA labels are assigned to matched compounds using data from both the Drug Repurposing Hub and ChEMBL. Compounds labelled as kinase inhibitors were downloaded before a literature review excluded inhibitors which would likely elicit little to no consistent phenotypic effect in U2OS cells. Quality control was then devised to exclude samples where low quality images were taken by the microscope, this was done using three quality factors - blur, saturation and focus. The remaining samples were included within our kinase inhibitor dataset.

Different approaches to data processing were taken for each data modality, detailed in Figure S10 under the individual modalities. The image-based profiles represent the profiles output by the CellProfiler software which are included with the raw images in the cpg0016 JUMP dataset s3 bucket. Different models were tested for each modality before the best-performing processing techniques and model architectures were combined to form the CVF Fusion model architecture.

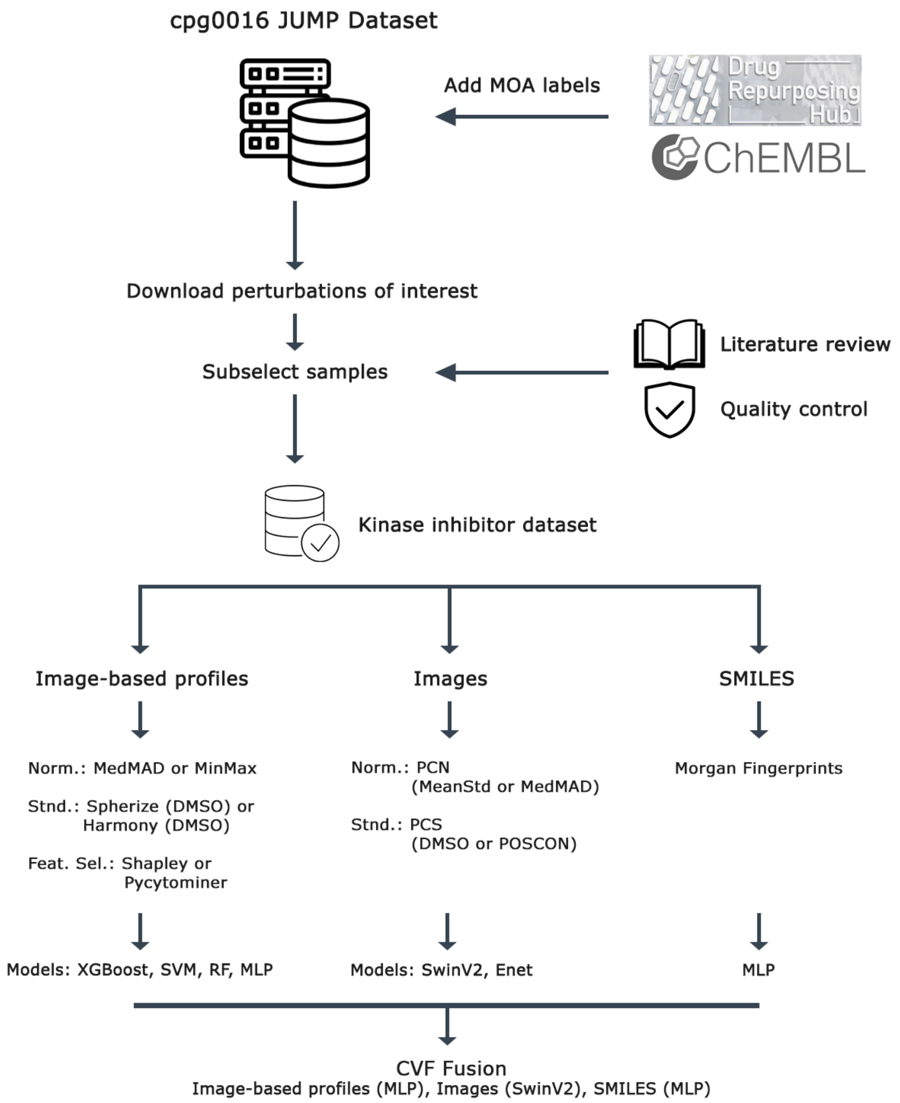

Supplemental Information, Figure S11. Workflow overview, related to Figure 2.

An overview of the workflow used in this study, describing the dataset selection process, subsequent different processing and modelling approaches, and eventual combination of the optimal approaches into the CVF Fusion architecture.

REFERENCES

1. Seal, S., Yang, H., Trapotsi, M.A., Singh, S., Carreras-Puigvert, J., Spjuth, O., and Bender, A. (2023). Merging bioactivity predictions from cell morphology and chemical fingerprint models using similarity to training data. *Journal of Cheminformatics*, 15, 56. 10.1186/s13321-023-00723-x.
2. Liu, Y., Cox, S.R., Morita, T., and Kourembanas, S. (1995). Hypoxia Regulates Vascular Endothelial Growth Factor Gene Expression in Endothelial Cells. *Circulation Research*, 77, 638–643. 10.1161/01.RES.77.3.638. https://www.ahajournals.org/doi/full/10.1161/01.RES.77.3.638. Publisher: American Heart Association.
3. Pal Singh, S., Dammeijer, F., and Hendriks, R.W. (2018). Role of Bruton's tyrosine kinase in B cells and malignancies. *Molecular Cancer*, 17, 57. 10.1186/s12943-018-0779-z. https://doi.org/10.1186/s12943-018-0779-z.
4. Neshat, M.S., Raitano, A.B., Wang, H.G., Reed, J.C., and Sawyers, C.L. (2000). The Survival Function of the Bcr-Abl Oncogene Is Mediated by Bad-Dependent and -Independent Pathways: Roles for Phosphatidylinositol 3-Kinase and Raf. *Molecular and Cellular Biology*, 20, 1179–1186. https://www.ncbi.nlm.nih.gov/pmc/articles/PMC85238/.
5. Chen, P.H., Chen, X., and He, X. (2013). Platelet-derived growth factors and their receptors: structural and functional perspectives. *Biochimica et biophysica acta*, 1834, 2176–2186. 10.1016/j.bbapap.2012.10.015. https://www.ncbi.nlm.nih.gov/pmc/articles/PMC3612563/.
6. Traves, P.G., Murray, B., Campigotto, F., Galien, R., Meng, A., and Paolo, J.A.D. (2021). JAK selectivity and the implications for clinical inhibition of pharmacodynamic cytokine signalling by filgotinib, upadacitinib, tofacitinib and baricitinib. *Annals of the Rheumatic Diseases*, 80, 865–875. 10.1136/annrheumdis-2020-219012. https://ard.bmj.com/content/80/7/865. Publisher: BMJ Publishing Group Ltd Section: Rheumatoid arthritis.
